# Empowering Women in Thoracic Surgery: Findings From a Cross-Sectional Survey of the ESTS Women in General Thoracic Surgery Academy Survey

**DOI:** 10.1093/icvts/ivag248

**Published:** 2026-09-29

**Authors:** Melek Ağkoç, Hanna Tihanyi, Sara Lo Torto, Sara Sakowitz, Mara B Antonoff, Nuria Novoa Valentin, Paula Duarte D’Ambrosio, Isabelle Opitz, René H Petersen, Giulia Veronesi, Cristina Viola, Sri Subramaniam, Cecilia Pompili

**Affiliations:** Department of Thoracic Surgery, Erzurum City Hospital, 25240 Erzurum, Turkey; Department of Thoracic Surgery, National Institute of Oncology Budapest-Semmelweis University, 1122 Budapest, Hungary; Department of Thoracic Surgery, Humanitas Research Hospital, 20089 Rozzano, Milan, Italy; Department of General Surgery, Massachusetts General Hospital, Boston, MA 02114, United States; Department of Thoracic and Cardiovascular Surgery, MD Anderson Cancer Centre, Houston, TX 77030, United States; Department of Thoracic Surgery, Puerta de Hierro University Hospital, Madrid 28222, Spain; Department of Thoracic Surgery, Faculdade de Medicina, Universidade de Sao Paulo, Hospital Clinicas de Sao Paulo, São Paulo 01246-903, Brazil; Department of Thoracic Surgery, University Hospital Zurich and University of Zurich, Zurich 8091, Switzerland; Department of Cardiothoracic Surgery, Copenhagen University Hospital, Rigshospitalet, Copenhagen 2100, Denmark; Department of Thoracic Surgery, IRCCS San Raffaele Scientific Institute, Milan 20132, Italy; Department of Thoracic Surgery, Norfolk and Norwich University Hospital, Norwich NR4 7UY, United Kingdom; Medical Affairs, Medtronic, Watford WD18 8WW, United Kingdom; Institute for Clinical and Applied Health Research, University of Hull, Hull HU6 7RX, United Kingdom

**Keywords:** women surgeons, gender equity, mentorship, leadership development

## Abstract

**Objectives:**

The Women in General Thoracic Surgery (WGTS) Academy, organized by the European Society of Thoracic Surgeons (ESTS), supports early and mid-career women thoracic surgeons through mentorship, leadership development, and clinical immersions in a gender-equitable environment.

**Methods:**

We conducted a prospective cross-sectional survey-based evaluation of all fellows participating in the WGTS Academy between 2023 and 2025. The survey addressed the value of face-to-face meetings, simulation training, and clinical immersions, as well as professional connections, challenges faced, and suggestions for future improvements.

**Results:**

Twenty-nine women Academy fellows were invited, and 24 completed the survey (82.8%). Thirteen participants (54.2%) worked in academic or university hospitals. Face-to-face meetings during clinical immersions were highly valued (62.5% rating them 10/10). Simulation training was considered valuable by 23 participants (95.8%). The most appreciated topics were mentorship, leadership, robotic surgery, gender-related discussions, and opportunities to interact with women leaders. Participants identified key benefits such as international networking, mentorship, professional development, emotional support, and friendships. Regarding clinical impact, 8 participants (33.3%) reported changes in their practice, and 11 (45.8%) planned to implement changes. Six participants (25.0%) had already assumed leadership roles, and 7 participants (29.2%) were engaged in research. Overall, 23 participants (95.8%) would recommend the Academy, and 21 (87.5%) expressed willingness to participate in future activities.

**Conclusions:**

The WGTS Academy was highly valued by participants, who reported perceived benefits related to mentorship, networking, leadership development, and clinical practice. Participants identified opportunities for further program development, particularly in hands-on training and research support.

## Graphical abstract

**Figure:**
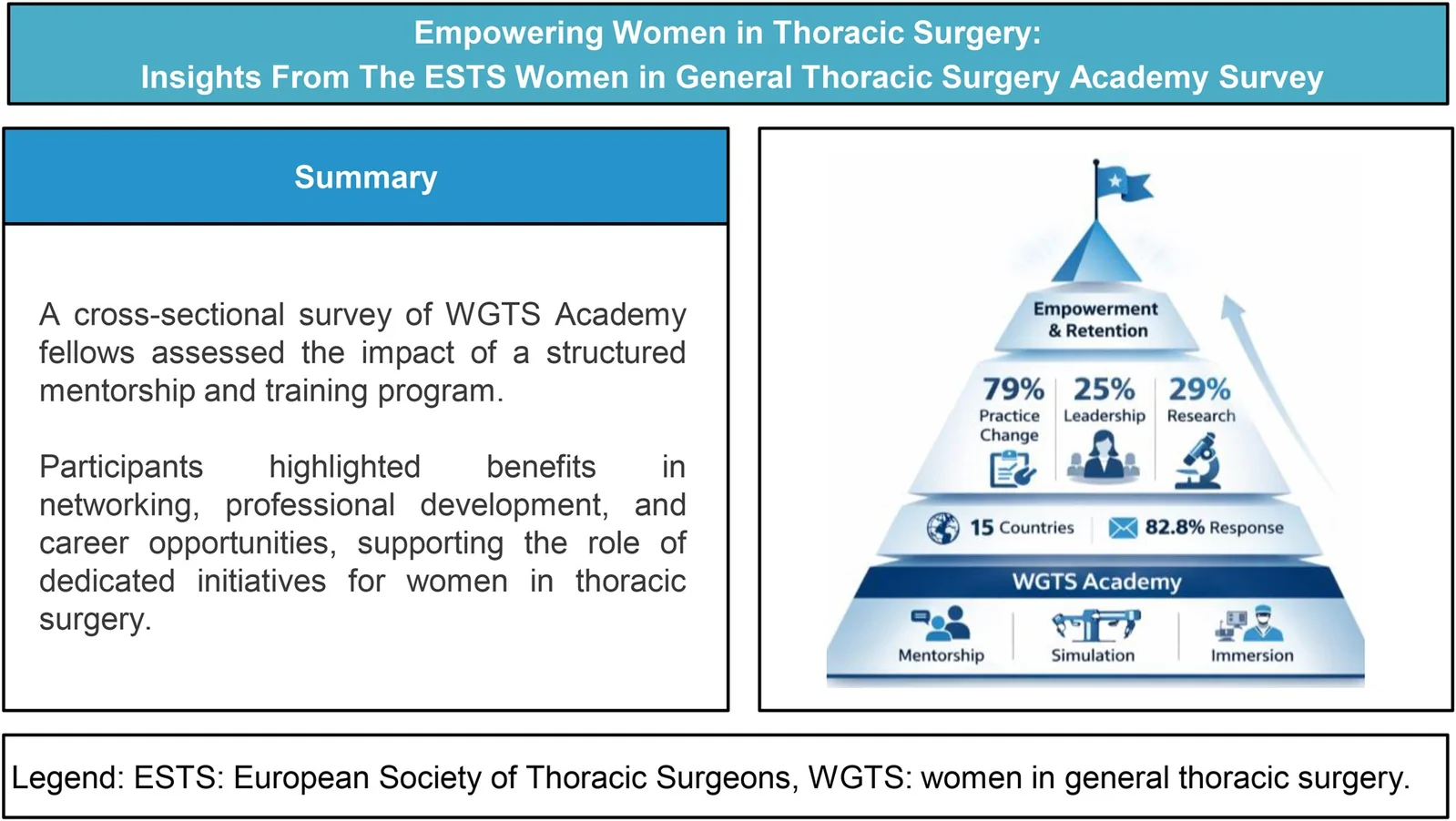

## INTRODUCTION

The worldwide situation of women’s underrepresentation in thoracic surgery continues to be a challenging and recognized problem that has an impact on recruitment, retention, and diversity of the workforce in the long run.^1^ Although there has been a gradual progress in recent years, the limited retention of women surgeons in many regions reflects the existence of barriers due to the structure of programs, limited access to a mentor, and gender biases in the training atmosphere.^1^^,^^2^ The small number of women in leadership positions, academic and association roles, keeps the situation perpetual, resulting in fewer future women surgeons and a reduced diversity of perspectives in the field that are necessary for its progress.^3^ Mentorship, leadership development, and exposure to role models are key factors known to influence career trajectories in surgical training.^4^ Studies have shown that women benefit from being under a structured mentoring system and a professional support network, which helps increase their confidence, resilience, and research and leadership aspirations.^4^ Programs designed to create a community, provide hands-on training opportunities, and address gender-specific problems such as work-life balance, implicit bias, and lack of representation have the potential not only to uplift the professional development but to secure the continuing presence of women surgeons in thoracic surgery as well.

The Women in General Thoracic Surgery (WGTS) Academy is an educational program established by the European Society of Thoracic Surgeons (ESTS) to support women early- and mid-career surgeons by providing mentorship from experienced women thoracic surgeons and offering structured learning opportunities through in-person networking events, clinical immersions, and live webinars (**Figure 1**). The program primarily targets surgeons at this stage of professional development, as it represents a particularly critical period for building mentorship relationships, international networks, academic collaborations, and leadership development in cardiothoracic surgery. The objective of the present study was to conduct a prospective evaluation of the WGTS Academy, assessing the participants’ experiences and quantifying the perceived benefits of this project on career development, networking, and challenges in clinical practice.

**Figure 1.**
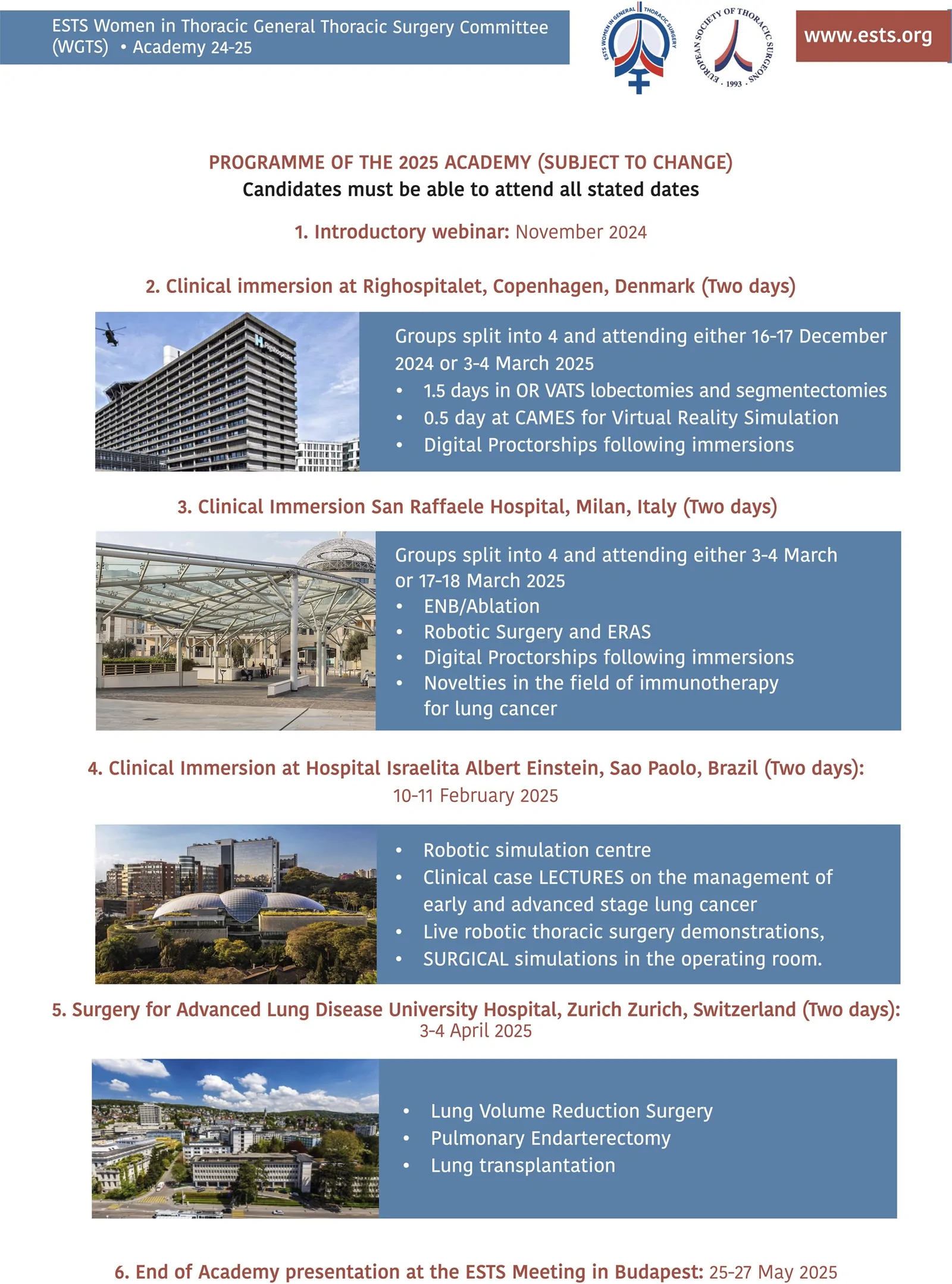
Program of the WTS Academy in 2025

## METHODS

### Study design and setting

We conducted a prospective cross-sectional survey-based evaluation of fellows participating in the WGTS Academy between 2023 and 2025. The WGTS Academy is an international educational and mentorship program organized by the ESTS, combining webinars, simulation-based training, face-to-face meetings, and clinical immersion experiences hosted at international thoracic surgery centres. To date, the Academy has graduated 29 women thoracic surgeons from multiple countries worldwide.

The program combines multiple educational components delivered throughout the fellowship period. Face-to-face meetings consist of in-person networking and educational sessions held during international thoracic surgery meetings and WGTS Academy events, providing opportunities for mentorship, leadership discussions, and peer interaction. Clinical immersion experiences involve short-term observerships at host thoracic surgery centres, allowing participants to observe clinical decision-making, multidisciplinary discussions, operating room activities, and institutional practices. Simulation-based training includes hands-on sessions focused on surgical skills development and exposure to emerging technologies, including minimally invasive and robotic thoracic surgery platforms. In addition, fellows participate in webinars and academic sessions covering topics related to thoracic surgery, leadership, mentorship, career development, and gender equity.

### Participants and data collection

Data collection was performed in June 2025. All fellows who participated in the WGTS Academy between 2023 and 2025 were eligible and invited to complete the survey via e-mail. The survey remained open for 3 weeks, with one reminder e-mail sent after 2 weeks. Because the WGTS Academy included a limited number of fellows, all eligible participants were invited to participate, and no formal sample size calculation was performed.

Survey responses were collected anonymously through the Qualtrics online platform (Qualtrics). To reduce potential sources of bias, all eligible fellows were invited to participate, a reminder e-mail was sent to maximize the response rate, and responses were collected anonymously without identifiable information to minimize social desirability and reporting bias while protecting participant confidentiality.

### Survey development and data collection

The questionnaire was developed by the WGTS Academy steering committee to evaluate participants’ experiences and the perceived impact of the program. Survey items were generated based on the educational objectives of the Academy and refined through expert consensus among faculty members. The survey was designed specifically for program evaluation purposes and was not formally validated.

The questionnaire consisted of 21 items, including Likert-scale, multiple-choice, and open-ended questions (**Material S1**). Domains evaluated included mentorship, leadership development, simulation-based training, clinical immersions, professional networking, career development, challenges encountered during program participation, and suggestions for future improvements. All study variables were obtained from participants’ self-reported questionnaire responses.

The primary outcomes of interest were participants’ perceived value of WGTS Academy activities, including face-to-face meetings, simulation-based training, and clinical immersions. Secondary outcomes included perceived impact on clinical practice, leadership development, research involvement, professional networking, and recommendations for future program development.

### Ethics and consent

This study consisted of an anonymous, voluntary, non-interventional survey evaluating participants’ experiences within an educational and mentorship program. No patient data, identifiable personal information, or sensitive data were collected. Participation was voluntary, and completion of the questionnaire implied informed consent. Responses were collected anonymously through Qualtrics and analysed in a de-identified format. Given the anonymous, voluntary, and non-interventional nature of the survey, no formal ethics committee review was sought.

### Statistical analysis

Survey responses were analysed using descriptive statistics. Categorical variables are presented as frequencies and percentages. Likert-scale responses were treated as ordinal variables and summarized using response distributions and summary measures as appropriate. No composite scores were generated, and all survey items were analysed individually. Open-ended responses were analysed using conventional thematic content analysis. Two investigators independently reviewed the responses, identified recurrent themes, and resolved discrepancies through discussion and consensus. Since individual responses could include multiple ideas, they were assigned to more than 1 theme where appropriate. The frequency of each theme was recorded. The most commonly reported themes and related subthemes are presented, along with representative quotations from participants.

Given the exploratory nature of the study and the small sample size, no inferential statistical testing was performed. Missing data were reported for individual survey items when applicable, and percentages were calculated based on the number of respondents providing valid responses to each question.

This study is reported in accordance with the Strengthening the Reporting of Observational Studies in Epidemiology (STROBE) guidelines. The completed STROBE checklist is provided in **Material S2**.

## RESULTS

### Participant characteristics

Of the 29 eligible fellows, 24 completed the survey (response rate 82.8%) and were included in the final analysis (**Table 1**). Five invited fellows did not respond. No missing data were observed among completed questionnaires. Thirteen respondents (54.2%) worked in academic or university hospitals, and 10 (41.7%) were employed in public or governmental institutions. Participants represented 15 countries across Europe, South America, North Africa, and the Middle East (**Figure 2**).

**Figure 2.**
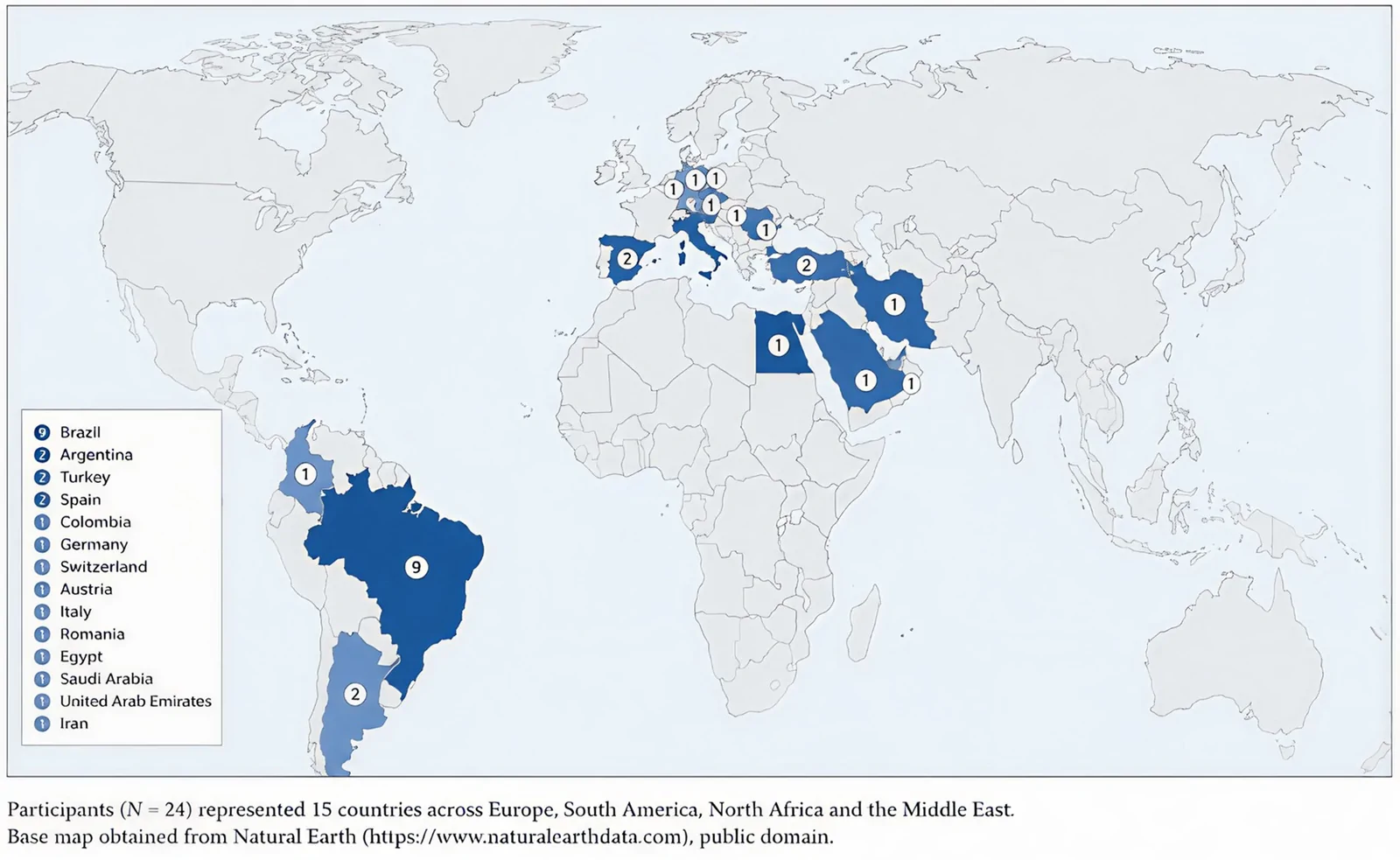
Participants (*N* = 24) Represented 15 Countries across Europe, South America, North Africa, and the Middle East. Base Map Obtained from Natural Earth (https://www.naturalearthdata.com), Public Domain

**Table 1.** Participant Characteristics

Characteristic	*n*(%)
Total respondents	24 (82.2)
Countries represented	15
Academic/University hospital	13 (54.2)
Public/Government institution	10 (41.7)

### Program evaluation

A summary of quantitative responses across the main questionnaire domains is presented in **Table S1**. Face-to-face interactions during clinical immersions received consistently high evaluations, with 15 participants (62.5%) assigning the maximum score (10/10) and an overall mean score of 9.13 ± 1.32. Simulation-based training was also highly valued, with 23 respondents (95.8%) reporting a positive educational impact and 10 (41.7%) rating this component as extremely valuable.

When participants were asked to identify the most valuable educational content, leadership, mentorship and sponsorship emerged as the most frequently selected topics (37.5%, *n* = 9) (**Table 2**). Exposure to new technologies and clinical approaches, including robotic surgery and simulation training, was highlighted by 33.3% of respondents (*n* = 8), while 25.0% (*n* = 6) identified networking and peer-to-peer exchange as the most valuable aspects of the program.

**Table 2.** Thematic Analysis of Responses Open-Ended Responses

Category	Main themes identified	Representative quotation
Participant expectations	International networking and collaboration (*n* = 15)	*Engage with female colleagues, build a strong network of female thoracic surgeons around the globe, collaborate and support other members’ research projects, promote women equity…*
	Professional and surgical development (*n* = 13)	*I hope to continue growing within this international network—sharing knowledge, building collaborative research or training opportunities…*
	Mentorship and career inspiration (*n* = 4)	*I would like to get to know the many accomplished and inspiring female thoracic surgeons around the world. I truly need their experience, guidance, and support.*
Most valued topics	Leadership, mentorship and sponsorship (*n* = 9)	*The discussions around gender bias, leadership development, and work-life balance in thoracic surgery were especially meaningful. Hearing real-life experiences from established female surgeons helped normalize challenges and fostered a sense of belonging.*
	Innovative surgical techniques and technologies (*n* = 8)	*LVRS, robotic bronchoscopic procedures, and segmentectomy were all highly valuable. In addition to these, the case discussions we had with our mentors … were also extremely insightful.*
	Networking and shared experiences (*n* = 6)	*Building a connection and network with other fellows and faculty.*
Perceived benefits of participation	International networking and collaboration (*n* = 22)	*Exchanging knowledge between different countries and continents.*
	Professional learning and skill development (*n* = 16)	*Exposure to high level surgery.*
	Career development and peer support (*n* = 13)	*Visibility and Empowerment—Being part of a female-focused group in a male-dominated field has been empowering, boosting confidence and giving visibility to women’s voices in thoracic surgery.*
Clinical practice changes	Implementation of minimally invasive and robotic techniques (*n* = 2)	*I am more confident performing VATS independently.*
	Changes in clinical decision-making and patient management (*n* = 2)	*Reduced drainage suction, implemented ERAS aspects in our daily practice.*
	Implementation of institutional change (*n* = 4)	*The Academy gave me greater confidence in advocating for myself, in proposing changes within my team, and in bringing forward ideas related to structured training, simulation, and inclusive team leadership.*
Future educational needs	Advanced surgical training (*n* = 8)	*Upper airways surgery. or Thoracic trauma.*
	Research methodologies/Scientific writing (*n* = 6)	*Writing articles and how to do clinical investigation…*
	Professional development and leadership (*n* = 8)	*Encouraging young doctors to become a thoracic surgeon. How to make this specialty attractive to both women and men.*

Abbreviations: ERAS, enhanced recovery after surgery; VATS, video-assisted thoracic surgery.

### Perceived benefits and professional impact

The most frequently reported benefits of participation were international networking and collaboration (91.6%, *n* = 22), followed by learning new techniques (66.6%, *n* = 16), and career development and emotional support (54.1%, *n* = 13) (**Figure 3A**).

**Figure 3.**
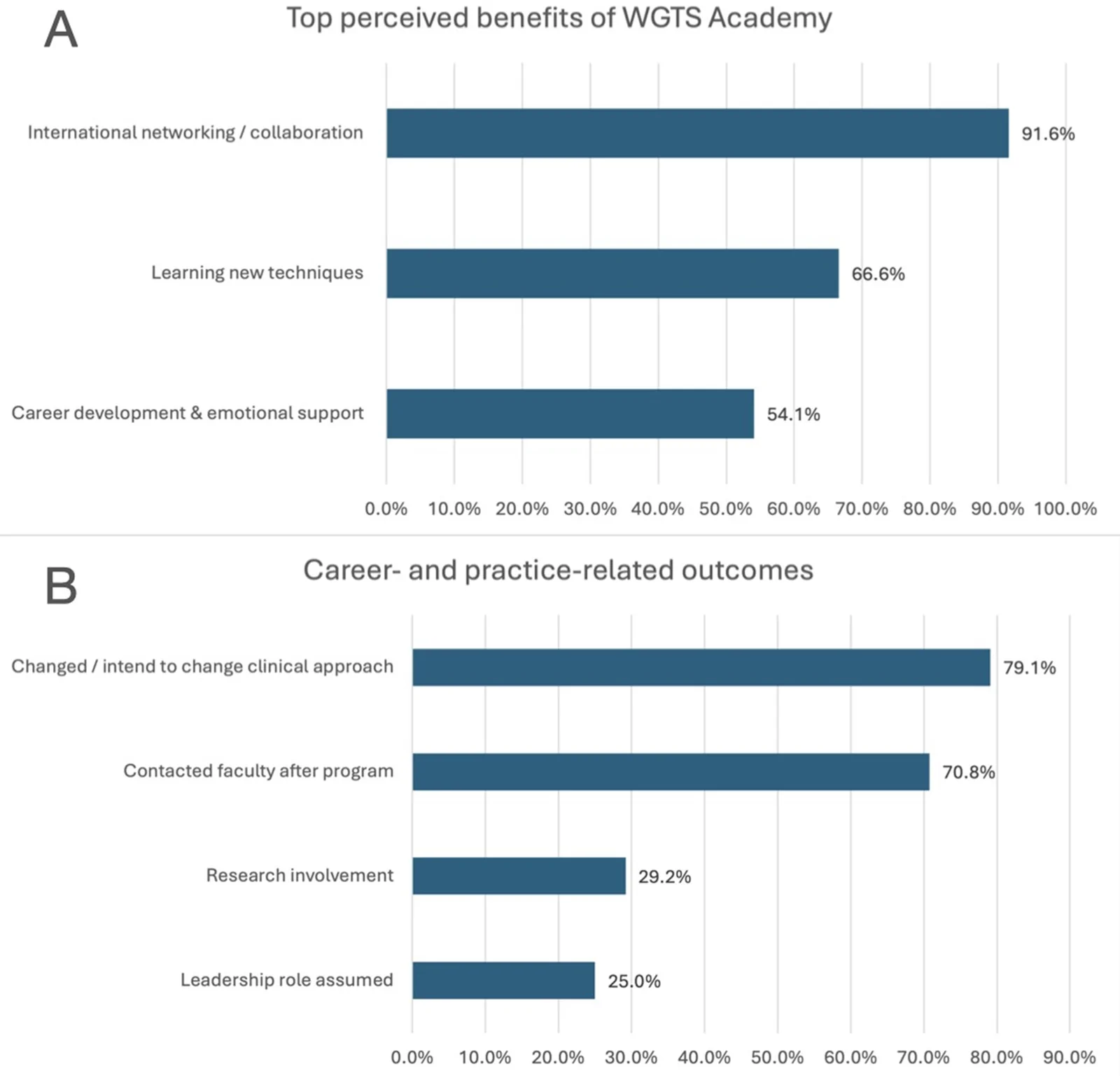
Impact of the WGTS Academy. (A) Top perceived benefits of the WGTS Academy. (B) Career- and practice-related outcomes reported by participants.

Beyond perceived educational benefits, participants reported tangible professional outcomes following program participation (**Figure 3B**). Eight participants (33.3%) reported that participation in the WGTS Academy had changed their clinical practice, while 11 (45.8%) reported no change and 5 (20.8%) were unsure. Among those reporting changes, the most commonly described impacts included increased confidence in minimally invasive thoracic surgery, implementation of enhanced recovery after surgery (ERAS) principles, improvement of perioperative management strategies, and greater involvement in education and mentorship within their institutions (**Table 2**). Seventeen participants (70.8%) reported maintaining professional contact with faculty members after program completion. Seven respondents (29.2%) reported involvement in research projects, while 6 (25.0%) reported having assumed leadership positions at the institutional, national, or international level following participation in the Academy.

The results of the open-ended response analysis, including recurring themes related to participants’ initial expectations and future educational needs, are also presented in **Table 2**. Four participants (17.4%) reported challenges in participating in the immersion program. The most commonly reported barriers were logistics (*n* = 2), visa-related issues (*n* = 2), financial constraints (*n* = 1), and other challenges (*n* = 2), including communication with the program coordinator and the close scheduling of immersions, which complicated travel arrangements and obtaining time away from work.

## DISCUSSION

This study provides a cross-sectional evaluation of participant experiences within the ESTS WGTS Academy. The main findings were the high-perceived value of mentorship, international networking, simulation-based training, and clinical immersion experiences. Participants also reported perceived benefits related to professional development, including leadership opportunities, research involvement, and changes or intended changes in clinical practice. These findings should be interpreted within the broader context of the persistent underrepresentation of women in cardiothoracic surgery and the ongoing need for structured professional development opportunities within the specialty.^5^^,^^6^

Mentorship and networking emerged as the most highly valued components of the program. More than 90% of respondents identified international networking and collaboration as a major benefit of participation, while leadership, mentorship, and sponsorship were among the most frequently selected educational topics. These findings are consistent with previous reports highlighting the importance of structured mentorship programs and professional support networks for women in surgery.^7–9^ In highly specialized surgical fields, where women remain underrepresented and opportunities for peer support may be limited, mentorship initiatives may help reduce professional isolation, increase visibility, and facilitate access to sponsorship and career development opportunities.^8^^,^^9^

The Women in Thoracic Surgery (WTS) Mentorship Program in North America has previously demonstrated the value of targeted mentorship and networking structures for women cardiothoracic surgeons.^7^ In the European setting, despite well-described gender disparities and the persistent underrepresentation of women in leadership positions, structured programs with reported outcomes remain limited.^6^ The WGTS Academy contributes to this landscape by combining mentorship, international networking, simulation-based training, clinical immersion experiences, and leadership-focused educational content within a single structured program. The international composition of the cohort further provides opportunities for cross-country collaboration, exchange of clinical experience, and exposure to different healthcare systems. Participants consistently identified these components as valuable, suggesting that the program addresses professional needs that may not be consistently met through traditional training pathways.

Participants also reported perceived professional benefits following program participation. One quarter of respondents reported having assumed leadership positions at the institutional, national, or international level, while nearly one-third reported involvement in research projects. In addition, almost 80% indicated that they had changed or intended to change aspects of their clinical practice following participation in the Academy. Although these findings are based on self-reported perceptions and do not establish causality, they suggest that participants perceived the program as relevant to both their professional development and daily clinical activities. Similar observations have been reported by the WTS Mentorship Program, which highlighted the value of targeted mentorship and networking opportunities in supporting career advancement and increasing the visibility of women within cardiothoracic surgery.^7^

The high value attributed to international networking and emotional support is also noteworthy. Professional isolation, limited access to role models, and lack of representation remain recognized challenges for women in highly specialized and traditionally male-dominated surgical fields.^10^ Previous studies have demonstrated persistent disparities in academic visibility, research productivity, and representation in senior authorship and leadership positions among women cardiothoracic surgeons.^11^ In this context, the WGTS Academy provides a dedicated environment for peer and senior mentorship, international networking, and professional exchange. The strong emphasis placed on these elements by participants suggests that such opportunities address important professional needs that may not be consistently available within local training environments.

The qualitative findings provide important insights into areas for future program development. Participants consistently identified hands-on surgical training, research methodology, and scientific writing as priorities for expansion, suggesting that educational needs extend beyond mentorship and networking alone. These findings are particularly relevant given the well-described disparities in academic productivity, professional recognition, and career advancement experienced by women surgeons.^2^^,^^11^^,^^12^ In addition, the interest in educational content addressing work–life balance and gender equity reflects challenges that continue to influence career progression and retention within surgery.^13–15^

Practical barriers identified by participants, particularly financial and visa-related constraints, also warrant consideration. As the WGTS Academy continues to expand internationally, addressing these logistical challenges may improve accessibility and facilitate participation from a broader range of geographical and socioeconomic backgrounds. Collectively, these findings may help inform the future evolution of the program, supporting the development of a more comprehensive curriculum that integrates technical training, academic development, leadership skills, and professional support.

Several limitations should be considered when interpreting these findings. First, this was a cross-sectional survey conducted at a single time point in a relatively small cohort (*n* = 24), limiting assessment of long-term career progression, retention, and sustained professional impact. In addition, the questionnaire was developed specifically for program evaluation and was not formally validated. Participation in both the WGTS Academy and the survey was voluntary, introducing the possibility of selection bias, as respondents may have had a particular interest in mentorship, leadership development, and professional networking. Additionally, participants represented a highly selected cohort of women thoracic surgeons enrolled in an international mentorship program, which may limit the generalizability of the findings to other surgical specialties, different training settings, or professional development initiatives.

Furthermore, all outcomes were based on self-reported perceptions and experiences and are therefore subject to self-reporting, response, and social desirability biases. Participants may also have been biased to provide more favourable evaluations because they directly benefited from the program and may have felt a sense of gratitude or loyalty towards the Academy, its mentors, and the society, and this could potentially lead to overestimation of program satisfaction and perceived impact. Although the response rate was high, non-responders may have held different views, potentially introducing non-response bias. Finally, the absence of baseline assessments and a comparator group preclude evaluation of changes over time and limits attribution of observed outcomes exclusively to participation in the WGTS Academy. Nevertheless, the high response rate and consistency of findings across respondents support the internal validity of the survey results.

## CONCLUSION

Participants highly valued the WGTS Academy and reported positive perceptions regarding mentorship, networking, leadership development, and clinical practice. Participants identified opportunities for further program refinement, particularly through expanded hands-on training, research support, and educational content addressing leadership and gender equity. Incorporating these priorities may further strengthen the program and support its role in fostering mentorship, professional development, and international collaboration among WTS.

## Supplementary Material

ivag248_Supplementary_Data

## Data Availability

The data supporting the findings of this study are available from the corresponding author upon request.

## Abbreviations

ESTS: European society of thoracic surgeons
ERAS: enhanced recovery after surgery
VATS: video-assisted thoracic surgery
WGTS: Women in general thoracic surgery
WTS: Women in thoracic surgery
