# Supplementary material for: Empowering Women in Thoracic Surgery: Findings From a Cross-Sectional Survey of the ESTS Women in General Thoracic Surgery Academy Survey
Source: Interdiscip Cardiovasc Thorac Surg. 2026 Sep 29;41(11):ivag248. doi: 10.1093/icvts/ivag248 (PMC13730561; doi:10.1093/icvts/ivag248)
Supplement: Supplementary file 1 [file ivag248_supplementary_data.zip › Supplementary Material 1. .docx]

**EUROPEAN SOCIETY OF THORACIC SURGEONS (ESTS) WOMEN IN GENERAL THORACIC SURGERY (WGTS) ACADEMY EXPERIENCE SURVEY**

**Q1. What is your affiliation (Institution/Hospital/Organization)?** *Response options: Multiple choice (single answer)*

- Academic / University Hospital
- Public / Government Hospital or Institution
- Private Hospital or Clinic

**Q2. Which country do you practice in?**

*Response options: Multiple choice (single answer)*

▼ Afghanistan (1) ... Zimbabwe (198)

**Q3. How valuable was the face to face meeting in Brazil / Milan / Copenhagen / Zurich / London? Rate 1-10 (10 being most valuable)**

*Response options: 11-point Likert-type scale ranging from 0 to 10.*

**Q4. Have you reached out to any of the presenters you met in Brazil / Milan / Copenhagen / Zurich / London?**

*Binary response (Yes/No)*

**Q5. Is simulation training valuable in the curriculum for a thoracic trainee?**

*Binary response (Yes/No)*

**Q6. If YES: How valuable? Rate 1-10 (10 being most valuable)**

*Response options: 11-point Likert-type scale ranging from 0 to 10.*

**Q7. Are there any specific topics that you feel should have been raised in this Academy that was not addressed?**

*Response format: Open-ended response*

**Q8. What was the most valuable topic you felt was raised in this forum?**

*Response format: Open-ended response*

**Q9. What do you want to achieve from being a member of this group?**

*Response format: Open-ended response*

**Q10. Did you stay in touch with any new members following the group being set up?**

*Binary response (Yes/No)*

**Q11. What have you felt been the top three benefits of being in this group?**

*Response format: Three open-ended response*

**Q12. Are there any suggestions to improve the communication within the group?**

*Response format: Open-ended response*

**Q13. Any suggestions for webinars that specifically raise issues or learning that relates to you?**

*Response format: Open-ended response*

**Q14. Any other topics you would like to be addressed in future Academy?**

*Response format: Open-ended response*

**Q15. Are you willing to be more involved in future ESTS WGTS activities?**

*Binary response (Yes/No)*

**Q15.2. If YES, please specify!**

*Response format: Open-ended response*

**Q16. Have you had any issue in participating the immersions?**

*Binary response (Yes/No)*

**Q17. If YES; what kind of challenges did you face?**

*Response format: Multiple-choice (multiple answers allowed)*

- Logistics
- Financial
- Annual leave / Permissions
- Visa
- Other, please specify:

**Q18. Have you changed your clinical approach at your institution after having attended the ESTS WGTS Academy?**

*Response options: Multiple choice (single answer)*

- Yes
- Not yet, but I know what I would like to change.
- No.

**Q18.2. If YES, please specify!**

*Response format: Open-ended response*

**Q19. Have you had any leadership role at your Institution/Nationally/Internationally because of having attended the ESTS WGTS Academy?**

*Response options: Multiple choice (single answer)*

- Not yet.
- In the future I will.
- Yes, I already had.

**Q20. Have you been involved in any research project at your Institution/Nationally/Internationally because of having attended the ESTS WGTS Academy?**

*Response options: Multiple choice (single answer)*

- Not yet.
- I know about a project that I will be involved in.
- Yes, I already had.

**Q21. Would you recommend the WGTS Academy to your colleagues?**

*Response options: Multiple choice (single answer)*

- Yes
- No
- Maybe
