# Supplementary material for: Empowering Women in Thoracic Surgery: Findings From a Cross-Sectional Survey of the ESTS Women in General Thoracic Surgery Academy Survey
Source: Interdiscip Cardiovasc Thorac Surg. 2026 Sep 29;41(11):ivag248. doi: 10.1093/icvts/ivag248 (PMC13730561; doi:10.1093/icvts/ivag248)
Supplement: Supplementary file 1 [file ivag248_supplementary_data.zip › Suppl. Table 1.docx]

# Supplementary Table 1. Summary of quantitative questionnaire responses (N = 24 unless otherwise indicated)

| Domain | Item | n | % | Denominator |
| --- | --- | --- | --- | --- |
| **Perceived value of program components** | Face-to-face meetings during clinical immersions — maximum rating (10/10) | 15 | 62.5% | N = 24 |
|  | Face-to-face meetings — mean rating (0–10 scale) | 9.13 ± 1.32 (mean ± SD) | — | N = 24 |
|  | Simulation-based training — positive educational impact | 23 | 95.8% | N = 24 |
|  | Simulation-based training — rated “extremely valuable” | 10 | 41.7% | N = 24 |
| **Most valued educational content** | Leadership, mentorship, and sponsorship | 9 | 37.5% | N = 24 |
|  | New technologies / robotic surgery / simulation | 8 | 33.3% | N = 24 |
|  | Networking and peer-to-peer exchange | 6 | 25.0% | N = 24 |
| **Perceived benefits of participation** | International networking and collaboration | 22 | 91.6% | N = 24 |
|  | Learning new techniques | 16 | 66.6% | N = 24 |
|  | Career development and emotional support | 13 | 54.1% | N = 24 |
| **Professional / clinical impact** | Change in clinical practice following participation | 8 | 33.3% | N = 24 |
|  | No change in clinical practice | 11 | 45.8% | N = 24 |
|  | Unsure whether practice changed | 5 | 20.8% | N = 24 |
|  | Maintained professional contact with faculty | 17 | 70.8% | N = 24 |
|  | Involvement in research projects | 7 | 29.2% | N = 24 |
|  | Assumed a leadership position (institutional/national/international) | 6 | 25.0% | N = 24 |
| **Overall program satisfaction** | Would recommend the Academy | 23 | 95.8% | N = 24 |
|  | Willing to participate in future activities | 21 | 87.5% | N = 24 |
| **Challenges / barriers to participation** | Reported a challenge during the immersion† | 4 | — | N = 24 |
|  | — Logistics | 2 | — | of 4 reporting challenges |
|  | — Visa-related issues | 2 | — | of 4 reporting challenges |
|  | — Financial constraints | 1 | — | of 4 reporting challenges |
|  | — Other (coordination/scheduling) | 2 | — | of 4 reporting challenges |

SD; standard deviation.
